# Basilar artery curvature increases the risk of posterior circulation infarction occurrence in patients without vertebrobasilar stenosis

**DOI:** 10.1007/s10072-022-06566-y

**Published:** 2022-12-24

**Authors:** Shugang Cao, Mingfeng Zhai, Jun He, Jian Wang, Tingting Ge, Qian Wu, Xiaoxing Ni, Ping Cui, Wen’an Xu, Mingwu Xia

**Affiliations:** 1 Department of Neurology, The Second People’s Hospital of Hefei, Hefei Hospital Affiliated to Anhui Medical University, No. 246 Heping Road, Hefei, 230011 China; 2 Department of Neurology, Fuyang People’s Hospital, Sanqing Road, Fuyang, 236000 China; 3 Department of Radiology, The Second People’s Hospital of Hefei, Hefei Hospital Affiliated to Anhui Medical University, No. 246 Heping Road, Hefei, 230011 China

**Keywords:** Stroke, Posterior circulation infarction, Basilar artery dolichoectasia, Basilar artery curvature

## Abstract

**Introduction:**

Limited cross-sectional or case–control studies have identified the relationship between basilar artery (BA) curvature and posterior circulation infarction (PCI). This study aimed to identify the influence of BA curvature severity on the risk of PCI occurrence in patients without vertebrobasilar stenosis through a prospective cohort study.

**Methods:**

In this study, we enrolled 171 patients with BA dolichosis but without vertebrobasilar stenosis. The BA geometric parameters were evaluated on MRA. The primary outcome was the occurrence of PCI, mainly referring to cerebellar and/or brainstem infarction. Cox proportional hazard models were used to detect possible predictors of PCI.

**Results:**

Among them, 134 (78.4%) patients were diagnosed with BA curvature, including 124 with moderate curvature and 10 with prominent curvature. The defined PCI occurrence was observed in 32 (18.7%) patients with a median follow-up time of 45.6 months. Cox proportional hazard analysis showed that BA prominent curvature (*HR* = 6.09; 95% *CI*: 1.36–27.28; *P* = 0.018) significantly increased the risk of PCI occurrence, and bending length (BL) was also significantly associated with PCI occurrence, with the adjusted *HR* per 1-mm increase of BL of 1.09 (95% *CI*: 1.01–1.18; *P* = 0.040). In the subgroup analysis stratified by age, BA prominent curvature was highly associated with PCI occurrence in patients aged > 61 years (*HR* = 11.76; 95% *CI*: 1.21–113.90; *P* = 0.033). Additionally, good antiplatelet therapy adherence could significantly reduce the risk of PCI occurrence.

**Conclusion:**

BA curvature may increase the risk of PCI occurrence, especially in elderly patients with prominent curvature. Improving adherence to antiplatelet therapy can help reduce the risk of PCI occurrence.

## Introduction

The vertebrobasilar system is the main blood supply for the posterior circulation, and stenosis or occlusion of the vertebrobasilar artery is naturally of great concern. Vascular curvature, a common morphological variant of the basilar artery (BA), has the most common alignment of a C-shape [1]. However, most BA curvatures do not meet the diagnosis of BA dolichoectasia (BADE) [1, 2] and are far less of a concern than BA stenosis.

Most previous studies considered that BA curvature is a vascular congenital variation and does not require special intervention. Recently, some studies have suggested that BA curvature is closely associated with the progression of BADE and the occurrence of posterior circulation infarction (PCI) [3, 4], possibly due to the resulting posterior circulation hemodynamic changes, abnormal blood perfusion, and subsequent atherosclerotic plaque formation [4–7]. However, these studies were cross-sectional or case–control studies, while prospective cohort studies on whether BA curvature is associated with the risk of PCI occurrence are lacking. In addition, most previous studies did not exclude the effect of BA stenosis on the occurrence of PCI. Theoretically, pronounced BA curvature could contribute to local atherosclerosis and even marked BA stenosis, but prior to the formation of vertebrobasilar stenosis, BA curvature primarily affects the pontine penetrating arteries, which in turn leads to the occurrence of PCI.

Our previous study indicated that BA dolichosis increased the risk of brainstem infarction recurrence in patients with acute pontine infarction, with one potential mechanism possibly being altered hemodynamics due to BA curvature, but this study also did not exclude patients with BA stenosis [2]. Therefore, it may be difficult to truly clarify the relationship between BA curvature and the risk of PCI occurrence. Whether there is a difference in the risk of PCI occurrence in patients with different degrees of BA curvature but without any vertebrobasilar stenosis remains unclear. Moreover, BA dolichosis does not necessarily have BA curvature, and BA curvature does not necessarily imply BA dolichosis. In this prospective cohort study, only patients with BA dolichosis without vertebrobasilar stenosis were selected to further investigate the relationship between the severity of BA curvature and the risk of PCI occurrence.

## Materials and methods

### Study population

In this prospective cohort study, we included patients with acute cerebral infarction (ACI) who were admitted to the Department of Neurology of our hospital from January 1, 2015 to December 31, 2019. Patients were consecutively enrolled if they were diagnosed with BA dolichosis but without vertebrobasilar stenosis on magnetic resonance angiography (MRA). We excluded patients with any causative stenosis in the vertebrobasilar artery, cardioembolism with evidence of embolic origin (e.g., atrial fibrillation, atrial flutter, and sick sinus syndrome), other determined etiologies (e.g., moyamoya disease or moyamoya syndrome, vasculitis, polycythemia vera, primary thrombocytosis, or dissection), S-shaped BA, BA aneurysms, intracranial tumors, or poor image quality affecting our measurements, patients without taking secondary stroke prevention medication for various reasons at discharge, and patients who refused to be followed up or died during hospitalization. This study was approved by the ethical committee of the Second People’s Hospital of Hefei. All patients or their guardians provided written informed consent.

### Clinical information assessment

Clinical-related data were acquired on the following factors: demographic data (including age and gender), and vascular risk factors such as current smoking, hypertension, diabetes mellitus, dyslipidemia, and ischemic heart disease. All patients with vascular risk factors had been previously diagnosed as such and/or were already taking medications for these conditions. Secondary prevention medications for ACI, including antiplatelet agents and statin therapy, were also collected at discharge and followed up throughout the study. Adherence to antiplatelet and statin therapy was estimated by calculating the proportion of days covered (PDC) during the entire follow-up period. The PDC is calculated as the total days of medication supplied divided by the duration of the follow-up period. If the PDC by antiplatelet agents or statin was above 80%, the patients were considered to have good adherence, otherwise poor adherence [8, 9].

### Imaging data

**Fig. 1:**
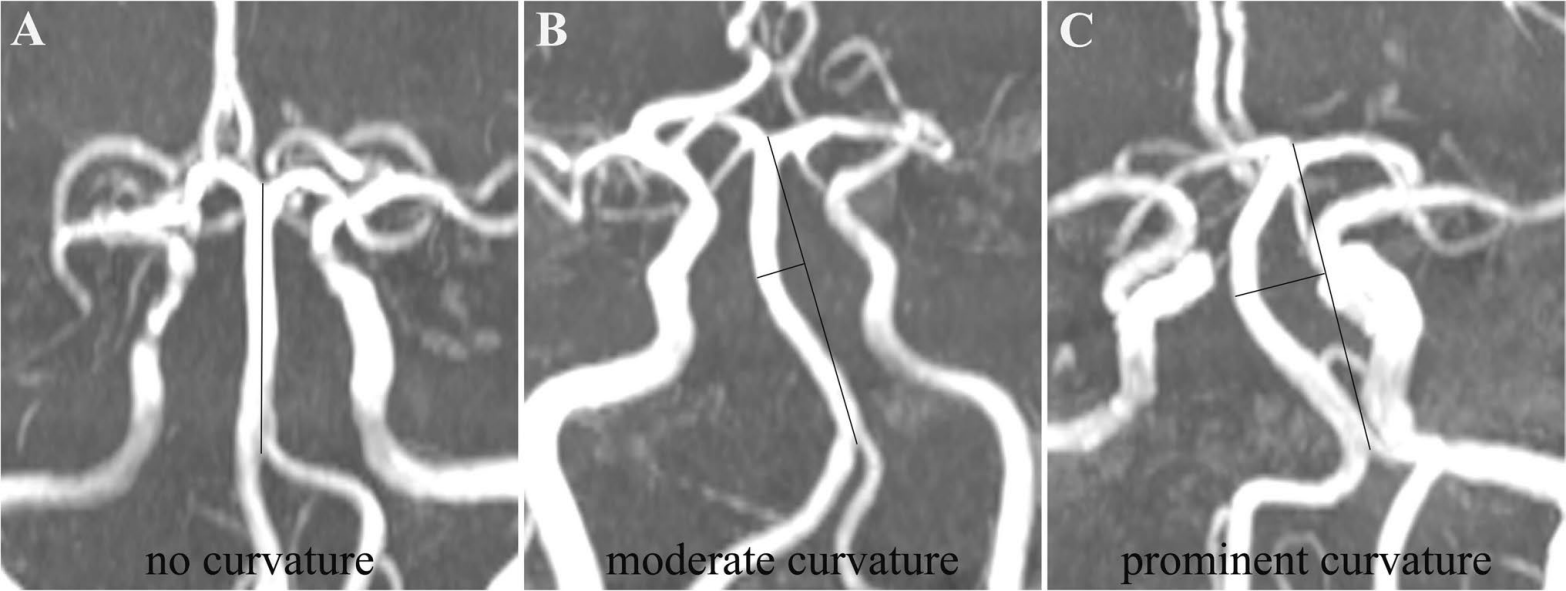
The severity of BA curvature was classified as no curvature (straight, **A**), moderate curvature (0 < BL ≤ 10 mm, **B**), and prominent curvature (BL > 10 mm, **C**)

Magnetic resonance imaging (MRI) and MRA were performed with a 1.5-Tesla scanner (Siemens Healthineers, Model: Avanto I class, Germany) on all patients. The DWI scan was conducted in the axial plane with a repetition time/echo time of 3400/102 ms and a slice thickness of 5 mm. We conducted MRA using a three-dimensional time of flight (TOF) sequence with a repetition time/echo time of 25/7 ms and a slice thickness of 0.6 mm. The geometric parameters of the BA and vertebral arteries (VAs) were evaluated in the posterior-anterior position. The methods for evaluating BA features, such as BA diameter, BA curve length, BA length (BAL), and bending length (BL), have been previously described in detail [10]. BA dolichosis was diagnosed with a BA curve length > 29.5 mm or BL > 10 mm. BA hypoplasia (BAH) was defined as BA diameter < 2 mm [1]. The BAL line was used to determine the position of the BA curvature [11]. The severity of BA curvature was classified into no curvature (straight), moderate curvature (0 < BL ≤ 10 mm), and prominent curvature (BL > 10 mm), as illustrated in Fig. 1. From the vertebrobasilar artery junction, a series of three measurements with 3-mm intervals at each side was taken, and the mean diameter served as the VA diameter [6]. The V4 segment hypoplasia (V4AH) of the VAs refers to a diameter of less than 2 mm. VA dominance (VAD) was defined as having a difference in the diameter of both VAs of at least 0.3 mm or as existing asymmetry with the VA connecting to the BA in a straighter fashion at the vertebrobasilar junction [5]. All vascular images were evaluated by two experienced neuroradiologists. When there was any disagreement, a radiologist with 10 years’ experience was consulted to resolve the issue.

### Follow-up assessments

Patients were followed up by periodic telephone interviews or clinic visits every 6 months by a trained neurologist blind to clinical and imaging data. The primary outcome was the occurrence of DWI-confirmed PCI, mainly referring to cerebellar and/or brainstem infarction other than temporo-occipital and (or) thalamic infarction here. If new stroke symptoms or signs appeared, they would be hospitalized and scheduled for imaging to determine whether acute PCI had occurred or whether they were stroke mimics. Infarct occurrence at other sites was not considered an endpoint event associated with morphological changes in the BA. The endpoint date for follow-up was December 31, 2021. The follow-up interval was defined as the time between enrollment and the initial occurrence of PCI, loss to follow-up, or death.

### Statistical analysis

All statistical analyses were performed with SPSS 22.0 package for Windows (SPSS Inc., Chicago, IL, USA). Continuous variables were tested for normality by the Kolmogorov–Smirnov test. Comparability between the three groups was analyzed using the Kruskal–Wallis test for non-normally distributed continuous variables or the ANOVA test for normally distributed continuous variables. Comparisons of categorical variables were performed using the Chi-square test for categorical variables. The Kaplan–Meier curve was performed to analyze the survival probability and compared using a two-sided log-rank test. Potential risk factors with a *P* value of < 0.1 in the univariate analysis were included in the multivariate Cox proportional hazard regression model with a forward stepwise procedure. Hazard ratio (*HR*) and 95% confidence interval (*CI*) were subsequently calculated. A two-sided *P* value of 0.05 was used as the threshold for statistical significance. GraphPad Prism 8.0 and Microsoft PowerPoint were used for graph drawing.

## Results

### Clinical data

**Fig. 2:**
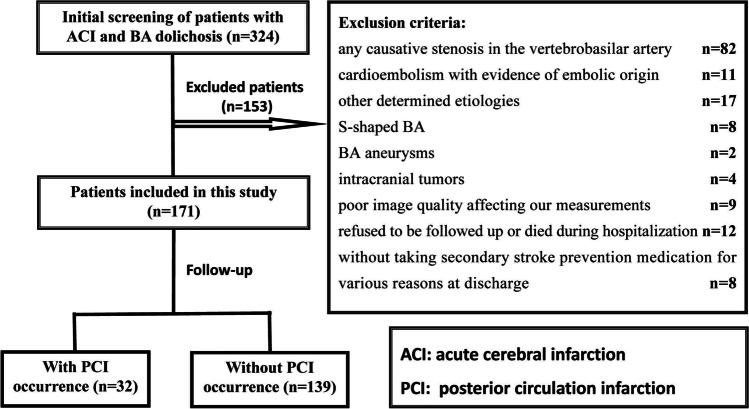
Study flowchart of the current study

As of December 31, 2019, a total of 324 patients with ACI combined with BA dolichosis were screened for this study; 171 patients were finally enrolled, including 112 patients with anterior circulation infarction and 59 patients with PCI, as shown in Fig. 2. Of these, 21 patients with BA dolichosis but no stenosis from our previous study were included in the present analysis [2]. The median age of onset was 61 years (ranging from 35 to 85 years), and 141 (82.5%) patients were male. Among them, 134 (78.4%) patients were diagnosed with BA curvature, including 124 of moderate curvature and 10 of prominent curvature, whereas the other 37 (21.6%) patients had no BA curvature. The median BL of patients with moderate curvature and prominent curvature were 6.18 (4.23 and 7.58) mm and 12.14 (10.25 and 13.31) mm, respectively. Additionally, 4 (2.3%) patients had BAH; 72 (42.1%) patients had V4AH; and 114 (66.7%) patients showed VAD. The baseline clinical and imaging data grouped according to BA curvature are presented in Table 1.

**Table 1:** Baseline data

Variable	No curvature (*n* = 37)	Moderate curvature (*n* = 124)	Prominent curvature (*n* = 10)	*P value*
**Age (years)**	61.0 (55.0, 67.0)	61.0 (53.0, 72.0)	68.5 (57.0, 82.0)	0.230
**Gender, *n* (%)**				0.098
Male	4 (10.8)	22 (17.7)	4 (40.0)	
Female	33 (89.2)	102 (82.3)	6 (60.0)	
**Risk factors, *n* (%)**				
Hypertension	26 (70.3)	88 (71.0)	6 (60.0)	0.766
Diabetes mellitus	12 (32.4)	36 (29.0)	4 (40.0)	0.734
Dyslipidemia	12 (32.4)	45 (36.3)	1 (10.0)	0.235
Ischemic heart disease	1 (2.7)	8 (6.5)	1 (10.0)	0.589
Smoking	15 (40.5)	57 (46.0)	4 (40.0)	0.809
**Good medication adherence, *n* (%)**				
Antiplatelet therapy adherence	32 (86.5)	115 (92.7)	9 (90.0)	0.493
Statin adherence	34 (91.9)	118 (95.2)	10 (100.0)	0.549
**BA features**				
BA diameter (mm)	3.34 (3.00, 3.53)	3.18 (2.83, 3.67)	3.36 (3.00, 3.62)	0.492
BAH (mm)	0 (0)	4 (3.2)	0 (0)	0.460
BAL (mm)	31.04 (30.30, 32.27)	30.89 (29.33, 33.46)	29.86 (27.35, 32.69)	0.466
BL (mm)	0 (0, 0)	6.18 (4.23, 7.58)	12.14 (10.25, 13.31)	< 0.001
**VA features, *n* (%)**				
VAD	23 (62.2)	84 (67.7)	7 (70.0)	0.798
V4AH	13 (35.1)	55 (44.4)	4 (40.0)	0.603

During the median follow-up time of 45.6 months (ranging from 3.1 to 78.2 months), 32 (18.7%) patients developed defined PCI with the average time from admission to occurrence of 34.4 ± 18.3 months. Of these, 3 occurred in the cerebellum, 25 in the brainstem (mainly in the pons), and 4 in the brainstem and cerebellum. Additionally, 10 patients experienced anterior circulation infarction or temporo-occipital and (or) thalamic infarction during follow-up, which was not considered an endpoint event, as it was probably unrelated to the morphological changes in the BA. The estimated 1-year, 3-year, 5-year, and 7-year incidence rates of PCI occurrence were 1.1%, 10.5%, 15.8%, and 18.7%, respectively. During the entire follow-up period, 9 patients were lost to follow-up, for a loss to follow-up rate of 5.3%, and 11 (6.4%) patients died. In detail, 1 patient died of recurrent ischemic stroke and related complications, 2 of esophageal cancer, 1 of acute coronary syndrome, 1 of traumatic cerebral hemorrhage, and 1 of falling injuries. The remaining 5 patients suffered sudden death and the cause of death was unknown. In addition, 3 patients had non-fatal cerebral hemorrhages (none in the brainstem), and 1 patient developed hemoptysis during the follow-up period.

### Cox proportional hazard analysis on the risk of PCI occurrence

**Fig. 3:**
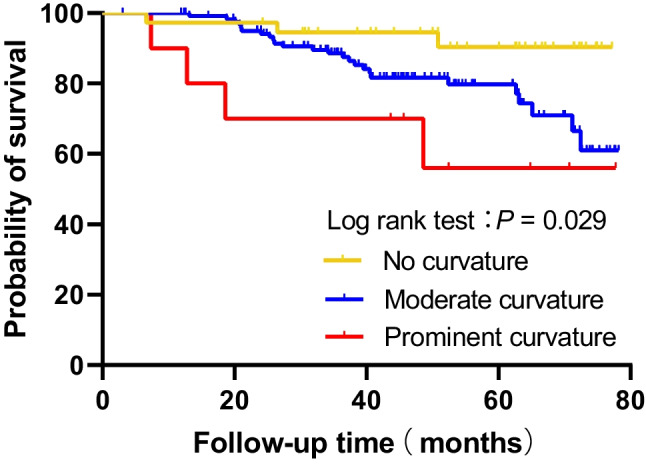
Kaplan–Meier curves estimating and comparing the survival probabilities of patients with no BA curvature, moderate curvature, and prominent curvature

The Kaplan–Meier curve showed a significantly higher rate of PCI occurrence in patients with BA moderate curvature and prominent curvature, compared to those with no BA curvature (adjusted *P* = 0.039 and 0.008, respectively, log-rank test) (Fig. 3). In univariate Cox proportional hazard model, BA prominent curvature (*HR* = 6.65; 95% *CI*: 1.49–29.79; *P* = 0.013) with no curvature as a reference and BL (*HR* = 1.10; 95% *CI*: 1.01–1.19; *P* = 0.026) were associated with an increased risk of PCI occurrence, while the difference looked clinically relevant even though not formally statistically significant in patients with moderate curvature (*HR* = 3.25; 95% *CI*: 0.98–10.80; *P* = 0.055) (Table 2). After adjusting age, BA diameter, and good antiplatelet therapy adherence with a *P* value of < 0.1 in the univariate analysis, BA prominent curvature (*HR* = 6.09; 95% *CI*: 1.36–27.28; *P* = 0.018) significantly increased the risk of PCI occurrence, while BA moderate curvature could also increase the risk of developing PCI, although not statistically significant (Table 3, model 1). We further introduced BL into Cox proportional hazard model as a continuous variable and found that BL was also significantly associated with PCI occurrence, with the adjusted *HR* per 1 mm-increase of BL of 1.09 (95% *CI*: 1.01–1.18; *P* = 0.040) (Table 3, model 2). In addition, age and good antiplatelet therapy adherence were also significantly associated with PCI occurrence in both multivariate Cox proportional hazard models (Table 3).

**Table 2:** Univariate Cox proportional hazard model for the risk of PCI occurrence

Variable	Number of cases (*n* = 171)	*HR* (95% *CI)*	*P value*
**Age**	61.0 (54.0, 71.5)	1.06 (1.02–1.10)	0.002
**Gender**			
Male	141 (82.5)	Ref	
Female	30 (18.5)	1.52 (0.68–3.39)	0.303
**Risk factors**			
Hypertension	120 (70.2)	1.73 (0.75–4.00)	0.203
Diabetes mellitus	52 (30.4)	1.67 (0.82–3.39)	0.155
Dyslipidemia	58 (33.9)	0.84 (0.41–1.76)	0.649
Ischemic heart disease	10 (5.8)	1.98 (0.60–6.51)	0.262
Smoking	76 (44.4)	1.08 (0.54–2.18)	0.828
**Good medication adherence**			
Antiplatelet therapy adherence	156 (91.2)	0.33 (0.13–0.88)	0.027
Statin adherence	162 (94.7)	0.54 (0.13–2.28)	0.402
**BA features**			
BA diameter	3.28 ± 0.65	0.56 (0.32–0.96)	0.035
BAH	4 (2.3)	3.10 (0.73–13.13)	0.124
BAL	30.94 (29.55, 33.19)	1.00 (0.92–1.09)	0.971
BL	3.24 (2.83, 3.65)	1.10 (1.01–1.19)	0.026
BA curvature			
No curvature	37 (21.6)	Ref	
Moderate curvature	124 (72.5)	3.25 (0.98–10.80)	0.055
Prominent curvature	10 (5.8)	6.65 (1.49–29.79)	0.013
**VA features**			
VAD	114 (66.7)	1.29 (0.60–2.79)	0.517
V4AH	72 (42.1)	0.91 (0.45–1.84)	0.794

**Table 3:** Multivariate Cox proportional hazard models for the risk of PCI occurrence

Models	*HR* (95% *CI*)	*P* value
**Model 1 (with BA curvature)**		
Age	1.06 (1.02–1.10)	0.002
Good antiplatelet therapy adherence	0.26 (0.10–0.71)	0.008
BA curvature		
No curvature	Ref	
Moderate curvature	3.07 (0.92–10.28)	0.068
Prominent curvature	6.09 (1.36–27.28)	0.018
**Model 2 (with BL)**		
Age	1.07 (1.03–1.11)	0.001
Good antiplatelet therapy adherence	0.30 (0.11–0.82)	0.019
BL	1.09 (1.01–1.18)	0.040

### Subgroup analysis

Further analysis showed that age grouped by median had interaction effects with BA curvature for the risk of PCI occurrence (*P* for trend = 0.002). The subgroup analysis stratified by age showed that BA prominent curvature was highly associated with PCI occurrence in patients aged > 61 years (*HR* = 11.76; 95% *CI*: 1.21–113.90, *P* = 0.033), but no significant association was found between BA curvature and PCI occurrence in patients aged ≤ 61 years.

## Discussion

In the present cohort study, we found that in patients with BA dolichosis but without vertebrobasilar stenosis, both BA prominent curvature and BL significantly increased the risk of PCI occurrence. In the subgroup analysis stratified by age, patients aged > 61 years with BA prominent curvature had a higher risk of PCI occurrence. In addition, good antiplatelet therapy adherence could significantly reduce the risk of PCI occurrence.

BA curvature is associated with congenital development and acquired factors and develops progressively with aging. The diagnostic criteria for BA curvature are MRI-based qualitative criteria [12] and MRA-based quantitative diagnostic criteria [1, 5, 7, 11]. Previous studies have suggested that BA curvature is mainly related to bilateral VA diameter difference and that the mechanical changes caused by the asymmetric VA flow lead to the varying flow force distribution on the BA vessel wall, thus inducing the BA gradually curving in the opposite direction from the larger VA [6]. Atherosclerotic plaques tend to develop in the context of BA curvature, and BA curvature progresses with increasing age and atherosclerotic risk factors [13, 14]. As a result, atherosclerosis and BA curvature form a vicious circle. Based on the above theory, the cross-sectional study by Zhang et al. suggested that vascular risk factors and BA curvature together increased the risk of pontine infarction [5]. Although patients with comorbid vascular risk factors were at higher risk of PCI, there were no statistically significant differences in this study, possibly related to the small sample size or formal secondary prevention after ACI.

The study by Zhang et al. showed that BL larger than 3.77 mm was an important risk factor for pontine infarction [5]. Our cohort study used semi-quantitative diagnostic criteria based on the MRA, with further grading of the severity of BA curvature and demonstrated that BA moderate and prominent curvature significantly increased the risk of PCI occurrence, compared to patients with no BA curvature. In addition, using BL, a quantitative indicator of the magnitude of BA curvature, we also found that for each 1-mm increase in BL, patients had a 1.088-fold increased risk of developing PCI. The potential mechanisms underlying the relationship between BA curvature and PCI are illustrated as follows. First, the mechanical and hemodynamic factors play a crucial role in the development of atherosclerotic plaques. Wall shear stress (WSS) is an important blood flow parameter that responds to the tangential friction generated by blood flow acting on the vascular endothelium. Early atherosclerotic plaques typically occur at sites with low or oscillatory WSS, such as the inner walls of tortuous arteries, yielding an atherothrombogenic environment [15, 16]. The study by Lee et al. showed that the distal region of the inner surface of the curved BA is a low WSS region, where the vessel wall is susceptible to damage and where the combination of vascular risk factors can activate a cascade of inflammatory cascades that promote plaque development and consequently block the entrance to the penetrating artery [17]. Brainstem infarcts occurred significantly more likely at the inner arc of the curved BA, suggesting a higher propensity for atherosclerotic thrombosis [17, 18]. Second, the hemodynamics of the BA bend is extremely complex with turbulence and vortex, and slower blood flow here renders the formation of mural microthrombus easily. Subsequently, shedding of the embolus leads to basilar artery perforator embolism. Third, BA curvature is likely to stretch and compress the BA perforating artery. As the magnitude of BA curvature increases, the probability of stretching and/or compression forces on the BA penetrating artery also increases, resulting in vasospasm and ischemic changes, which can easily lead to penetrating atherosclerosis and even stenosis or occlusion [5, 19]. Finally, the severity of BA curvature affects the blood perfusion of the posterior circulation. That is, the more severe the BA tortuosity, the more pronounced the hemodynamic disturbance. Specifically in CT perfusion imaging, as the deviation position and bifurcation height of the BA continued to increase, the relative cerebral blood flow (rCBF) and relative cerebral blood volume (rCBV) were shown to gradually decrease, while mean transit time (MTT) and time-to-peak time (TTP) gradually increased [4]. The more the posterior circulation perfusion is reduced, the greater the likelihood the PCI becomes.

Our further subgroup analysis found that elderly patients with BA prominent curvature were at a greater risk of PCI occurrence. Presumably, elderly patients have more vascular risk factors and are at higher risk of PCI in the presence of combined BA curvature. This could also explain why most patients with BA curvature are asymptomatic when they are young, and as they age and have increased risk factors for cerebrovascular diseases, these patients are at increased risk of developing PCI. Therefore, elderly patients with BA curvature should be closely monitored for the risk of PCI and given more stringent secondary prevention management. In addition, we found that good antiplatelet therapy adherence significantly reduced the risk of PCI occurrence. Thus, although most BA curvatures often do not require surgical intervention, these patients often have a comorbidity of multiple vascular risk factors or even multiple strokes, and may require more intensive management of antiplatelet agents to improve compliance with antiplatelet therapy and reduce the risk of PCI occurrence. Although cerebral hemorrhage occurred in 3 cases, all were in non-brainstem areas, which in turn suggests that antiplatelet therapy is relatively safe for patients with BA curvature or dolichosis and does not increase the risk of brainstem hemorrhage. If the patient has BA prominent curvature combined with BA ectasia, the risk of PCI and rupture hemorrhage may be both high. Endovascular treatment can even be indicated if necessary [20, 21].

In this prospective cohort study, we used semi-quantitative and quantitative criteria to evaluate BA curvature severity, and our results provided compelling support that the severity of BA curvature was significantly associated with the risk of long-term PCI occurrence. Nevertheless, this study also has the following limitations to highlight. First, although patients with vertebrobasilar stenosis were excluded from this study, the presence of plaque at the entrance to the perforating artery was not clarified by high-resolution MRI (HR-MRI). A previous study showed that among the 17 patients without BA stenosis on MRA, HR-MRI found a BA plaque in 7 patients [22]. Therefore, no vascular stenosis does not mean any atherosclerotic plaque formation. Second, this is a single-center cohort study based on hospital patients with ACI. The predictors of PCI occurrence may not accurately reflect the general population due to the limitations of case selection bias. Third, as this study selected DWI-confirmed site-specific PCI as the endpoint event, posterior circulation transient ischemic attacks or possible DWI-negative PCI were not included. That is because we could not confirm whether these symptoms were associated with defined PCI occurrence. In addition, some patients with PCI may be asymptomatic or less symptomatic and do not consult to have their DWI examinations completed. Therefore, these may underestimate the real PCI occurrence rate.

## Conclusion

In this prospective study, patients with BA prominent curvature may have a higher risk of PCI occurrence, especially in elderly patients, and therefore deserves more attention in clinical practice. Improving adherence to antiplatelet therapy can help reduce the risk of PCI occurrence.

## Data Availability

The datasets generated for this study will be made available by the authors to any qualified researcher.
